# Zearalenone Exposure Damages Skeletal Muscle Through Oxidative Stress and Is Alleviated by Glutathione, Nicotinamide Mononucleotide, and Melatonin

**DOI:** 10.3390/antiox14050528

**Published:** 2025-04-28

**Authors:** Dandan Li, Wei Fu, Jiyue Zhang, Yaqiu Lin, Xianrong Xiong, Jian Li, Yan Xiong

**Affiliations:** 1 Key Laboratory of Qinghai-Tibetan Plateau Animal Genetic Resource Reservation and Utilization, Ministry of Education, Southwest Minzu University, Chengdu 610041, China; lidandan@mnu.cn (D.L.); fuwei@swun.edu.cn (W.F.); zhangjiyue0416@163.com (J.Z.); yaqlin@163.com (Y.L.); xiongxianrong@163.com (X.X.); lijian@swun.edu.cn (J.L.); 2 College of Animal & Veterinary Sciences, Southwest Minzu University, Chengdu 610041, China; 3 Key Laboratory of Animal Science of National Ethnic Affairs Commission of China, Southwest Minzu University, Chengdu 610041, China

**Keywords:** zearalenone, toxicity, skeletal muscle, antioxidant, muscle fiber

## Abstract

Zearalenone (ZEN), a mycotoxin, is toxic to skeletal muscle, and the solution to alleviate its damage remains unknown. Here, we analyzed the toxic effect of ZEN on muscle and the mitigation of antioxidants (GSH, NMN, and melatonin) for this toxicity. The results showed that 0.02 mmol/L ZEN inhibited myoblast viability and myogenic differentiation, accompanied by reducing *Type I* and *Type IIA* and increasing *Type IIX* myofibers. Antioxidants (NMN with 0.5 mmol/L, GSH with 1 mmol/L, and melatonin with 1 mmol/L) rescued these phenotypes. Mice that were delivered 3 mg/kg body weight (BW)/day of ZEN by gavage for 35 days exhibited a similar trend of muscle fiber composition, but the gavage of antioxidants (NMN with 500 mg/kg BW/day, GSH with 300 mg/kg BW/day, and melatonin with 100 mg/kg BW/day) abolished this phenotype. Mechanistically, ZEN treatment increased ROS production, resulting in oxidative stress, mitochondrial dysfunction, and, subsequently, myofiber changes. Additionally, ZEN indirectly contributed to its damage, decreasing the abundance of *Lactobacillus* at the genus level and increasing *Streptococcus* sp. at the species level, which was associated with lactic acid production. Antioxidants partially rescued this microbiota composition. This study explores ZEN toxicity effects and alleviation of antioxidants, which provides new insights and attenuation solutions for ZEN damage to skeletal muscle. However, the underlying molecular mechanism of ZEN and antioxidants in the skeletal muscle still needs to be explored.

## 1. Introduction

Mycotoxin contamination is widespread in the world and has become a global concern, which can lead to significant economic losses in agriculture and seriously threaten the safety of food and the health of humans [1]. ZEN, also known as F-2 toxin, is a kind of mycotoxin that has been classified as a Group 3 carcinogen by the International Agency for Research on Cancer (IARC), primarily produced by *Fusarium graminearum* and *Fusarium culmorum* in the moldy corn, sorghum, wheat, and their products of cultivation area [2]. Extensive research reported that animals were exposed to ZEN, leading to reproductive toxicity in the sow [3] and immunotoxicity or cytotoxicity in various tissues of other animals, including the liver [4] and kidney [5]. The skeletal muscle is the largest locomotor organ, protein reservoir, and primary glucose metabolism site, accounting for ~40% of individual body weight [6]. Myofibers are the basic structural units of skeletal muscle, which are roughly divided into fast (*Type IIA*, *Type IIB*, and *Type IIX*) and slow (*Type I*) muscle fibers, respectively, with glycolytic and oxidative metabolic characteristics [7]. For humans, the composition and transformation of myofiber types are linked to individual normal physiological function and exercise capacity [8], while a higher proportion of slow-twitch fibers would improve meat quality for the animals [9]. Currently, the specific damage caused by ZEN exposure to skeletal muscle and muscle fiber composition is largely unknown.

Recently, several works provided evidence for high concentrations of ZEN detected in the muscle tissues when the animals are exposed to ZEN [10,11], which could be carried over in animal food products to further threaten human health. For instance, the highest concentrations of ZEN were observed in muscle tissue (12.033 ng/g) among the measured tissues of clinically healthy female wild boars fed naturally contaminated with ZEN at 50 mg/kg BW/day [10]. Consistently, ZEN residues are found in the leg muscle (0.069 µg/kg) of Hy-Line Brown laying hens fed a basal diet containing 60% moldy corn [11]. However, the deleterious effects of ZEN on skeletal muscle remain largely unknown. Damiano et al. found that ZEN natural exposure changed the morphology and myofiber diameter slightly in the muscles of wild boars from Southern Italy [12]. In addition, sows fed with ZEN-contaminated diets (2.77 mg/kg BW) from 35 to 70 days of gestation seem to be particularly sensitive and even decrease myofiber number and diameter in the muscle tissue of newborns and weaning piglets [13]. Thus, these studies suggested that animals exposed to ZEN would have damaged skeletal muscles. However, it was difficult to identify the direct pathological changes of skeletal muscle mediated by ZEN because of the complicated effects of the coexistence of multiple mycotoxins in moldy cereal crops and their products.

Previous studies have shown that oxidative stress is one of the main pathways through which ZEN toxicity causes impaired cell viability, mitochondrial dysfunction, etc. [14,15]. Therefore, antioxidant therapy may be a potential solution to alleviate the toxicity of ZEN to muscles. Nicotinamide mononucleotide (NMN), as another potential antioxidant, could increase oxidized nicotinamide adenine dinucleotide (NAD^+^) levels and rescue mitochondrial dysfunction in C2C12 cells and levator ani muscle (LAM) damaged by mechanical stress (MS) [16]. Glutathione (GSH) is a functional dietary supplement with detoxification [17] and antioxidant [18] functions, etc. Melatonin is a broad-spectrum free radical scavenger with strong antioxidant ability, which can directly or indirectly regulate the expression of cellular antioxidant enzymes, thereby enhancing the antioxidant capacity of cells [19]. Thus, GSH, NMN, and melatonin were selected as the candidate antioxidants to alleviate the damage to muscles mediated by ZEN.

Herein, we aimed to explore the toxicity of ZEN on skeletal muscle by measuring cell viability, myogenesis, and muscle fiber composition. Then, the underlying mechanism was revealed by measurement of ROS levels, mitochondrial function, and gut microbial composition. Moreover, we explored whether three antioxidants (GSH, NMN, and melatonin) could alleviate the muscle damage caused by ZEN in vitro and in vivo.

## 2. Materials and Methods

### 2.1. Cell Culture

C2C12 myoblast cell line (ATCC, Rockville, MD, USA) was cultured in a Growth medium consisting of Dulbecco’s modified Eagle medium (DMEM) (Gibco, C11995500BT, Shanghai, China), 10% Fetal bovine serum (FBS) (AlphaCell, 100061, Guangdong, China), and 1% penicillin-streptomycin (PS) (Biosharp, BL505A, Hefei, China). The C2C12 cells with a confluence of 80–90% were induced myogenic differentiation by a differentiation medium (DM) containing 2% horse serum (Solarbio, S9050, Beijing, China) and 1% PS. All the cell plates were maintained in a cell incubator at a constant temperature and humidity (37 °C, 5% CO_2_), and the medium was changed every two days. For the drug treatment, the stock solution of ZEN (HPLC ≥ 99.0%, Biopike, Waltham, MA, USA), NMN (HPLC ≥ 95.0%, Sigma-Aldrich, St. Louis, MO, USA), GSH (HPLC ≥ 98.0%, Sigma-Aldrich), and melatonin (HPLC: 99.95%, Selleck, Houston, TX, USA) were dissolved in DMSO and stored at −20 °C in the dark, which was used at a work solution concentration of ZEN with 0.02 mmol/L [20], NMN with 0.5 mmol/L [16], GSH with 1 mmol/L [21,22], and melatonin with 1 mmol/L [21,23], respectively. The Control (0.1% DMSO), ZEN, ZEN + NMN, ZEN + GSH, and ZEN + Melatonin treatment groups were designed. The drug is added to the C2C12 cells along with the DM, and collected 4 days after cell differentiation.

### 2.2. Animals and Study Approval

C57BL/6J female mice (8 weeks old) were purchased from the Chengdu Dossay Laboratory Animal Center. The mice were kept in a room with a temperature of 25 ± 1 °C, a humidity of 55 ± 5%, and a 12-h light cycle (light at 8:00 a.m.–8:00 p.m., dark at 8:00 p.m.–8:00 a.m.). Thirty female mice were randomly divided into 5 groups, including the Control (0.2 mL normal saline per day), ZEN (ZEN: 3 mg/kg BW/day [24]), ZEN + NMN (ZEN: 3 mg/kg BW/day + NMN: 500 mg/kg BW/day [24]), ZEN + GSH (ZEN: 3 mg/kg BW/day + GSH: 300 mg/kg BW/day [21,25]), ZEN + Melatonin groups (ZEN: 3 mg/kg BW/day + Melatonin: 100 mg/kg BW/day [21,26]). According to the drug solubility, ZEN, NMN, GSH, and melatonin were weighed and dissolved in DMSO and stored at −20 °C in the dark. Normal saline was added 30 min before gavage for dissolution, and each mouse was gavaged with 0.2 mL per day. Daily weight and food intake were recorded. After 35 days of gavage, the mice were sacrificed by cervical dislocation. The gastrocnemius (GAS), soleus (SOL), tibialis anterior (TA), and extensor digitorum longus (EDL) muscles of the hind legs in mice were collected and frozen with liquid nitrogen. All the tissues were stored in an ultra-low temperature freezer at −80 °C.

### 2.3. Cell Counting Kit 8 (CCK-8) Assay

C2C12 cells were cultured in 96-well plates, and six biological replicates were set up for each treatment group. For the CCK-8 assay, the cells were washed 2–3 times in PBS, added 100 μL of growth medium and 10 μL of CCK-8 (AbMole, M4839, Shanghai, China), and incubated in an incubator (37 °C) for 1 to 4 h sequentially. The full-band microplate reader (Perkin Elmer) measured the absorbance (450 nm) after 48 h of culture. The formula is cell viability = [A(dosing) − A(blank)]/[A(control) − A(blank)] × 100%. A (dosing): absorbance of cells, CCK-8 solution, and drug solution pores; A (control): absorbance of cells and CCK-8 solution pores; A (blank): absorbance of culture medium and CCK-8 solution pores.

### 2.4. Total RNA Extraction and Quantitative Real-Time PCR (RT-qPCR)

Collected C2C12 cells for 4 days of differentiation and leg muscles of mice after 35 days of gavage. The TRiZOL (Vazyme, R401-01, Nanjing, China) reagent was used to extract total RNA. The quality and concentration of RNA were detected by Nanodrop ND-1000 (Thermo Fisher, Waltham, MA, USA), and the OD_260_/OD_280_ with 1.8–2.0 was used to synthesize cDNA according to the manufacturer’s instructions (Prime-Script RT Master Mix, Takara, Dalian, China). Real-time quantitative PCR analysis was performed using a SYBR real-time PCR mixture (BioTeke, Beijing, China). The primer sequences for RT-qPCR are listed in Table 1. Each sample was repeated thrice, and the 2^−ΔΔCt^ method was used to calculate the relative mRNA levels.

### 2.5. Immunofluorescence Staining

The cells were fixed with 4% paraformaldehyde (Biosharp, BL539A, Hefei, China) and then washed with PBS three times. A total of 100 mM glycine was incubated for 10 min at room temperature and washed 3 times with PBS. A blocking buffer (2% BSA, 0.1% sodium azide, 0.2% Triton X-100, and 5% goat serum) was added to incubate cells for 45 min at room temperature. Added Primary antibody (MYHC, DSHB Hybridoma Product MF 20) for immunized cells overnight at 4 °C. After 3 washes of PBS, fluorescent secondary antibody, including Alexa FluorTM 568 Goat anti-rabbit IgG (H + L) (Invitrogen, A11011, Carlsbad, CA, USA) and Goat anti-mouse IgG H&L/FITC (Bioss, bs-0296G-FITC, Beijing, China), was incubated at room temperature for 1 h. The confocal microscope (Zeiss, LSM800, Oberkochen, Baden-Württemberg, Germany) was used to capture images. The fluorescence intensity (total fluorescence intensity divided by area), myotube fusion index (the ratio of the number of nuclei in the myotube to the total number), and total myonuclei from randomly selected areas of 3 biological replicates were analyzed by ImageJ software V1.8.0.112 [27].

### 2.6. Mito-Tracker Staining

The nucleus was stained with DAPI, and mitochondrial morphology was marked by Mito-Tracker Red staining (C1049B, Beyotime, Shanghai, China) according to the manufacturer’s instructions. Then, the cells were washed 3 times with PBS. Subsequently, the images were captured by a confocal microscope, and the red fluorescence density per area unit was measured by ImageJ software V1.8.0.112 [27]. There were 3 biological replicates in each group.

### 2.7. Reactive Oxygen Species (ROS) Evaluation by 2′,7′-Dichlorodihydrofluorescein Diacetate (DCFH-DA) Staining

The plate was seeded at a cell density of 3 × 10^5^ cells. Then, the cells were incubated with 10 μmol/L DCFH-DA (D6883; Sigma-Aldrich, St. Louis, MO, USA) in serum-free DMEM at 37 °C for 20 min. Cells were washed 3 times with serum-free DMEM to remove DCFH-DA that had not entered the cells adequately. The confocal microscope was used to capture images, and the ImageJ software V1.8.0.112 [27] was used to count fluorescence intensity.

### 2.8. Hematoxylin-Eosin (H&E) Staining

The GAS tissue was fixed in 4% paraformaldehyde for 24 h and dehydrated for 16 h. After routine embedding with paraffin, the tissue was cut into sections (5 µm). Then, the sections were placed in an oven (60 °C) for 1 h and were respectively dewaxed with xylene and ethanol and then washed with running water. The sections were further stained with hematoxylin for 5 min and placed under running water to remove residual color. Differentiated cells with 5% acetic acid for 10 s and washed with running water, then stained with 0.1% eosin for 1 min. After dehydration with alcohol, xylene is used to clear the tissue. Finally, the sections were sealed with neutral gelatin after drying. The confocal microscope was used to capture images, and ImageJ software V1.8.0.112 [27] was used to count the cross-sectional area.

### 2.9. Transmission Electron Microscopy (TEM)

The collected skeletal muscle was cut into less than 1 × 1 × 1 mm^3^ cubes, added 3% glutaraldehyde fixative solution within 1 min, rinsed in PBS, and then applied 1% osmium acid fixative solution for 2 h (pH = 7.3) sequentially. The samples were rinsed for 30 min with PBS and then dehydrated with acetone. Epoxy resin Epon 812 embedded tissue. An ultra-thin microtome machine was used to prepare 60–90 nm sections, and the sections were placed on a copper mesh with a supporting film. At room temperature, stained with uranyl acetate for 15 min. Then, the sections were stained with citrate for 2 min. A JEM-1400FLASH transmission electron microscope (JEOL Ltd., Tokyo, Japan) was used to capture images of copper meshes.

### 2.10. 16S rDNA Sequencing

The E.Z.N.A. Soil DNA Kit (Omega Bio-tek, Inc., Norcross, GA, USA) was used to extract genomic DNA from 15 mouse intestinal contents, and DNA quality and concentration were detected by Nanodrop 2000 (ThermoFisher Scientific, Inc., Waltham, MA, USA). Universal primers 338F (5′-ACTCCTACGGGAGGCAGCAG-3′) and 806R (5′-GGACTACHVGGGTWTCTAAT-3′) amplify the V3–V4 region of the bacterial 16S rRNA gene. A total of 8 bp barcode sequences were added to the 5′ ends of the upstream and downstream primers to distinguish different samples. Finally, universal primers with barcode sequences were synthesized and amplified on an ABI 9700 PCR instrument (Applied Biosystems, Inc., Foster City, CA, USA), and the size of the amplified target band was detected by 1% agarose gel electrophoresis. The Agencourt AMPure XP (Beckman Coulter, Inc., Pasadena, CA, USA) Nucleic Acid Purification Kit was used to automate the purification of PCR products, and the Caliper LabChip GX Touch HT (PerkinElmer, Inc., Waltham, MA, USA) measured the PCR product concentration and fragment size. The PCR products were then library constructed using the NEB Next Ultra II DNA Library Prep Kit (New England Biolabs, Inc., Ipswich, MA, USA) and purified with the Agencourt AMPure XP (Beckman Coulter, Inc., Pasadena, CA, USA) Nucleic Acid Purification Kit, Nanodrop 2000 (ThermoFisher Scientific, Inc., Waltham, MA, USA), Agilent 2100 Bioanalyzer (Agilent Technologies, Inc., Santa Clara, CA, USA) for library fragment size, and ABI StepOnePlus Real-Time PCR System (Applied Biosystems, Inc., Foster City, CA, USA) accurately quantified library concentration. Finally, the libraries were sequenced on the Illumina Miseq/Novaseq 6000 (Illumina, Inc., San Diego, CA, USA) platform with a sequencing strategy of PE250/PE300.

### 2.11. Statistics Analysis

The high-throughput 16S rDNA gene sequencing data were analyzed using the Qiime platform at Allwegene Company (Beijing, China). Each experiment was repeated thrice, and 3–6 biological replicates were set for each experimental group. All significant analyses were performed using SPSS 18.0 and GraphPad Prism 8.0 software. The data were presented as Mean ± SD and analyzed by One-way ANOVA; all differences were considered to be statistically significant at *p* < 0.05.

## 3. Results

### 3.1. ZEN Inhibits the Proliferation of Myoblast and Antioxidant Treatments Alleviate This Inhibitory Effect

**Figure 1:**
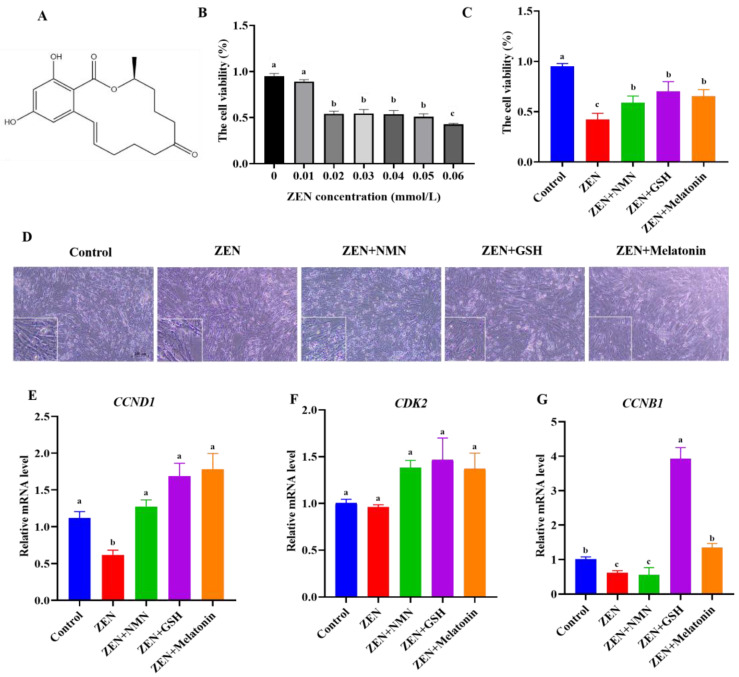
ZEN inhibits the cellular activity of myoblasts, and antioxidant treatment rescues this inhibition. (**A**) The chemical structural formula of ZEN, (**B**) CCK-8 assay detected the proliferation activity of myoblast treated by ZEN at concentrations of 0.01, 0.02, 0.03, 0.04, 0.05, and 0.06 mmol/L, respectively, (**C**,**D**) the cell viability (**C**) and cell morphology (**D**) (scale bar: 100 μm) were observed in Control, ZEN (0.02 mmol/L), ZEN + NMN (NMN with 0.5 mmol/L), ZEN + GSH (GSH with 1 mmol/L), and ZEN + Melatonin (melatonin with 1 mmol/L) groups, (**E**–**G**) the mRNA level of proliferation-related genes, including *CCND1* (**E**), *CDK2* (**F**), and *CCNB1* (**G**) in different treatment groups. Different letters (a–c) indicate significant differences (*p* < 0.05), and the same letters indicate no difference, *n* = 3.

The molecular formula of ZEN is C_18_H_22_O_5_ (Figure 1A), whose treatment concentration exceeded 0.02 mmol/L and significantly decreased the cell viability of C2C12 cells (Figure 1B), and this concentration was used in the further treatment of this study. Further, the CCK-8 assay showed a ~60% decrease in cell activity after ZEN treatment compared with the control group (Figure 1C). Compared with the ZEN treatment group, the combined treatment of antioxidants (ZEN + NMN, ZEN + GSH, and ZEN + Melatonin) all partially rescued cell viability and maintained cell morphology (Figure 1C,D). RT-qPCR detected the mRNA levels of proliferation-related genes, and data showed that cyclin d1 (*CCND1*) significantly decreased to 50% (Figure 1E) and cyclin b1(*CCNB1*) significantly decreased to 60% (Figure 1G) in the ZEN treatment compared with the control group. In contrast, the mRNA levels of *CCND1*, cyclin-dependent kinase 2 (*CDK2*), and *CCNB1* in the ZEN + NMN, ZEN + GSH, and ZEN + Melatonin groups were increased compared to those of ZEN treatment (Figure 1E–G). In addition, compared with the ZEN + NMN or ZEN + Melatonin groups, the ZEN + GSH group significantly promoted the expression of *CCNB1* (Figure 1G). Thus, these data indicated that ZEN inhibited the proliferation of myoblasts, and the inhibitory effect was alleviated after the combined treatment with NMN, GSH, and Melatonin to different extents, of which GSH had a better alleviation effect.

### 3.2. ZEN Suppresses the Differentiation of Myoblast and Antioxidant Treatment Rescues This Suppression Effect

**Figure 2:**
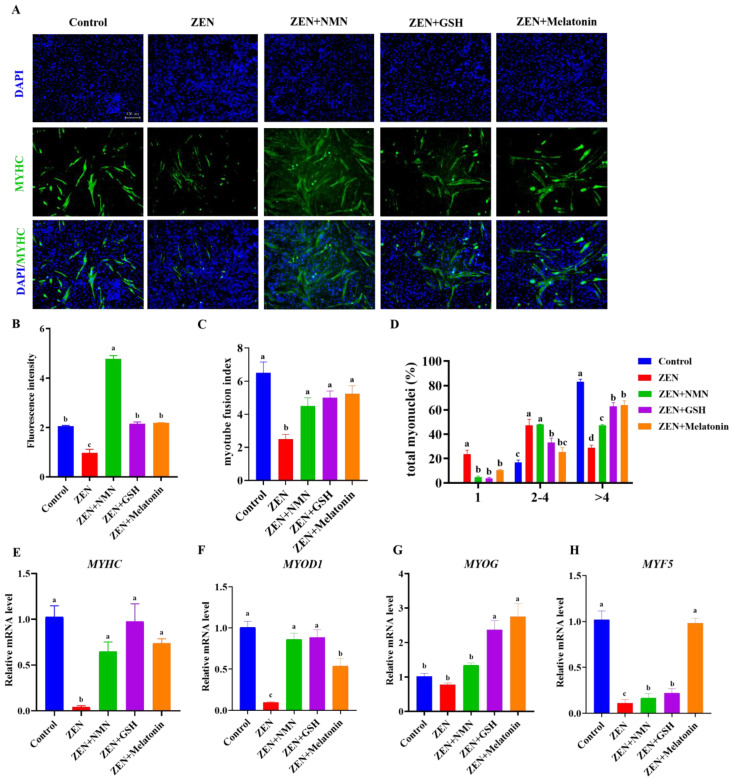
ZEN suppresses myogenic differentiation, and antioxidant treatment rescues this phenotype. (**A**) The images of MYHC immunofluorescence staining in the Control, ZEN, ZEN + NMN, ZEN + GSH, and ZEN + Melatonin groups, scale bar: 100 μm, (**B**–**D**) the count analysis of MYHC immunofluorescence intensity (**B**), the myotube fusion index (**C**), and the proportion distribution of different myo-nucleus (**D**), (**E**–**H**) the mRNA level of myogenic-related genes, including *MYHC* (**E**), *MYOD1* (**F**), *MYOG* (**G**), and *MYF5* (**H**) in the Control, ZEN, ZEN + NMN, ZEN + GSH, and ZEN + Melatonin groups. Different letters (a–d) indicate significant differences (*p* < 0.05), and the same letters indicate no difference, *n* = 3.

Next, the effect of ZEN on myogenic differentiation was measured, which is involved in mononuclear myoblasts fusing to form multinucleated myotubes [28]. The ZEN treatment significantly decreased myotube formation compared with that of the control (Figure 2A), indicated by the reduction of MYHC fluorescence intensity (Figure 2B, *p* < 0.05), myotube fusion index (Figure 2C, *p* < 0.05), and the proportion of >4 myonuclei myofiber (Figure 2D, *p* < 0.05). Interestingly, the inhibition of myotube formation caused by ZEN was rescued by the combination with the treatment of NMN, GSH, and melatonin (Figure 2B–D). At the mRNA level, ZEN treatment suppressed mRNA levels of myogenic markers, myosin heavy chain (*MYHC*) with a ~90% significant decrease (Figure 2E), myogenic differentiation 1 (*MYOD1*) with a ~80% significant decrease (Figure 2F), myogenin (*MYOG*) with a ~30% decrease (Figure 2G), and myogenic factor 5 (*MYF5*) with a ~75% significantly decrease (Figure 2H) compared with the control group, respectively. Compared with the ZEN group, the combined treatment of ZEN and antioxidants significantly up-regulated the expression levels of *MYHC*, *MYOD1*, *MYF5*, and *MYOG*, except for *MYOG* of the ZEN + NMN group (Figure 2E–H). Among the ZEN + GSH, ZEN + NMN, and ZEN + Melatonin groups, ZEN + Melatonin treatment significantly up-regulated most of the myogenic genes (Figure 2E–H). These results indicated that ZEN exposure inhibits myogenesis, which was rescued by antioxidant treatments (NMN, GSH, and Melatonin) to different extents.

### 3.3. ZEN Induces Slow-to-Fast Myofiber Shift and Antioxidant Treatment Rescues This Phenotype

**Figure 3:**
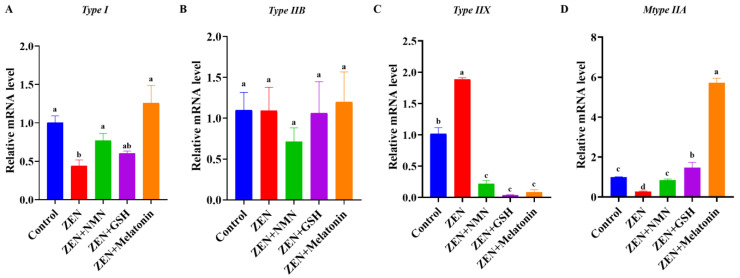
ZEN affected muscle fiber type-related gene levels, and antioxidant treatment rescued the myofiber change. (**A**–**D**) The mRNA levels of myofiber types of *Type I* (**A**), *Type IIB* (**B**), *Type IIX* (**C**), and *Type IIA* (**D**) in the aforementioned groups. Different letters (a–d) indicate significant differences (*p* < 0.05), and the same letters indicate no difference, *n* = 3.

Next, whether ZEN treatment induced the muscle fiber shift remains unknown. Oxidative myofibers are enriched in mitochondria, which can resist fatigue from long-term endurance exercise, while glycolytic myofibers rely more on glycolytic enzymes to produce energy quickly for rapid bursts of resistance movement [7]. Myofiber identification by RT-qPCR showed that the mRNA levels of oxidative myofiber *Type I* (*MYH7*) (Figure 3A) and oxidative myofiber *Type IIA* (*MYH2*) (Figure 3D) were significantly decreased in the ZEN treatment group compared with the control group. However, ZEN treatment increased the mRNA level of glycolytic myofiber *Type IIX* (*MYH1*), with ~2-fold significant changes compared to the control (Figure 3C, *p* < 0.05). For the antioxidant treatment, the gene expression of *Type I* and *Type IIA* in the ZEN + NMN, ZEN + GSH, and ZEN + Melatonin groups all showed a rescued effect compared with the ZEN group (Figure 3A,D). At the same time, the mRNA level of *Type IIX* significantly decreased to ~20%, ~4%, and ~10% of the control, respectively (Figure 3C). Furthermore, the ZEN + Melatonin group significantly increased the mRNA levels of *Type IIA* compared with the ZEN + NMN and ZEN + GSH groups (Figure 3D). Thus, it is concluded that ZEN affected the level of muscle fiber type-related genes, and antioxidant (NMN, GSH, and melatonin) treatments alleviated this change. Specifically, the rescued effect of melatonin was better compared with NMN and GSH.

### 3.4. ZEN Exposure Decreases Skeletal Muscle Myogenic-Related Gene Levels and Changes Myofiber Composition In Vivo

**Figure 4:**
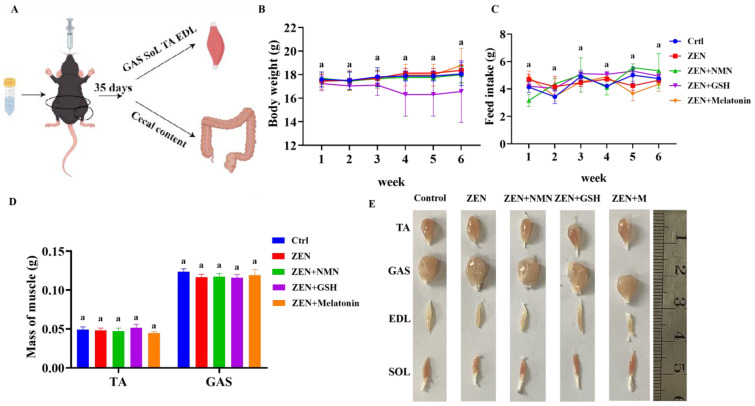
The mass of skeletal muscle is influenced by ZEN combined with antioxidant treatment. (**A**) A schematic diagram of gavage ZEN and antioxidant in mice was drawn by “Figdraw 2.0” software, (**B**,**C**) the body weight (**B**) and feed intake (**C**) of mice treated by ZEN and combined with antioxidants (NMN, GSH, and melatonin) for 6 weeks, (**D**,**E**) the mass (**D**) and images (**E**) of muscles (TA, tibialis anterior; GAS, gastrocnemius muscle; EDL, extensor digitorum longus; SOL, soleus) in the Control, ZEN, ZEN + NMN, ZEN + GSH, and ZEN + Melatonin groups. The same letter (a) indicates no difference, *n* = 6.

To explore the ZEN effect on skeletal muscle and screen an antioxidant to protect from ZEN damage in vivo, this mycotoxin and the antioxidants were delivered by gavage (Figure 4A). The results showed that the body weight (Figure 4B), feed intake (Figure 4C), skeletal muscle weight (Figure 4D), and muscle morphology (Figure 4E) were not significantly changed among the Control, ZEN, ZEN + NMN, ZEN + GSH, and ZEN + Melatonin groups.

**Figure 5:**
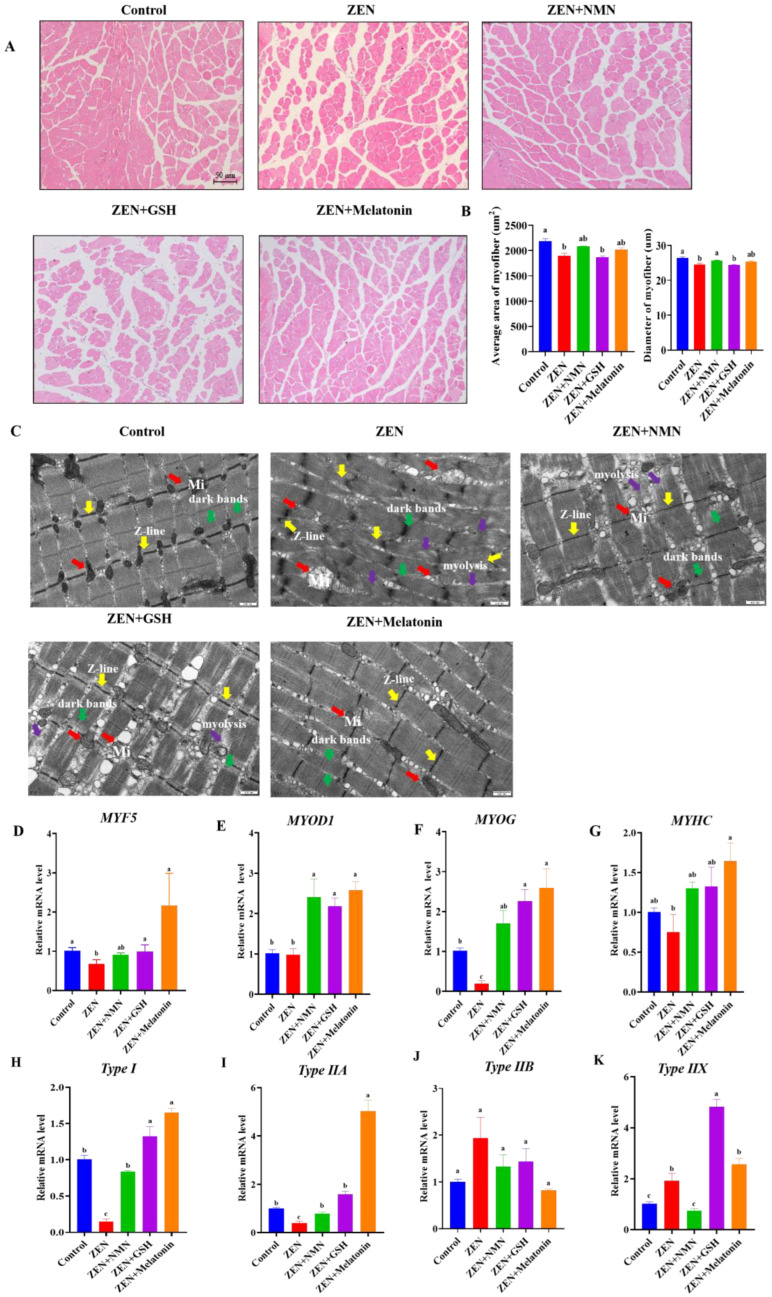
ZEN affected muscle fiber composition, and antioxidant treatment rescued the myofiber change in vivo. (**A**,**B**) The H and E staining (**A**) (scale bar: 50 μm) and average fiber area analysis (**B**) of GAS from Control, ZEN, ZEN + NMN, ZEN + GSH, and ZEN + Melatonin treated mice, (**C**) the ultrastructure of skeletal muscle observed by TEM, scale bar: 500 nm, (Red arrow: mitochondrial (Mi); Yellow arrow: Z-line; Green arrow: dark bands; Purple arrow: myolysis), (**D**–**K**) the mRNA level of myogenic genes (*MYF5* (**D**), *MYOD1* (**E**), *MYOG* (**F**), and *MYHC* (**G**)) and fiber type-specific genes (*Type I* (**H**), *Type IIA* (**I**), *Type IIB* (**J**), and *Type IIX* (**K**)) in the aforementioned groups. Different letters (a–c) indicate significant differences (*p* < 0.05), and the same letters indicate no difference, *n* = 6.

Next, the H&E staining and counting analysis found that the average area and diameter of myofibers in the ZEN group were significantly smaller than the control group (Figure 5A,B). Compared with the ZEN group, the average area and diameter of myofibers in the ZEN + Melatonin group were upward, and the average area of myofibers in the ZEN + NMN group was also increased, but the diameter of myofibers in the ZEN + NMN group was significantly increased (Figure 5B). In addition, the results of TEM showed that the ZEN group muscle had obvious lesions such as mitochondrial swelling, z-line irregularity, unclear light and dark bands, and myolysis compared with the control group (Figure 5C). However, the ZEN + NMN, ZEN + GSH, and ZEN + Melatonin groups all reduced lesions in comparison with ZEN treatment (Figure 5C). The expression levels of *MYF5* (Figure 5D) and *MYOG* (Figure 5F) were significantly lower in the ZEN group than those of the control, and *MYHC* (Figure 5G) was down-regulated. However, compared with the ZEN group, the mRNA levels of *MYF5*, *MYOD1*, *MYOG*, and *MYHC* were all partly or totally rescued in the ZEN + Melatonin, ZEN + GSH, and ZEN + NMN groups (Figure 5D–G). Among the three antioxidant treatment groups (ZEN + NMN, ZEN + GSH, and ZEN + Melatonin), the ZEN + Melatonin group had fewer lesions caused by ZEN (Figure 5C). From the above results, ZEN exposure damaged muscle in vivo, and antioxidants partly or totally rescued the deleterious effects, with melatonin having a more pronounced effect.

Furthermore, the mRNA levels of *Type I* (Figure 5H) and *Type IIA* (Figure 5I) were significantly lower, and that of *Type IIX* (Figure 5K) was significantly higher in the ZEN group than the Control group in vivo, which was consistent with the trend in vitro (Figure 3A–D). In the ZEN + NMN group, the mRNA levels of oxidative myofiber, including *Type I* (Figure 5H) and *Type IIA* (Figure 5I), were significantly higher, and myofiber *Type IIX* (Figure 5K) was significantly lower than in the ZEN group. In the ZEN + GSH group, *Type I* (Figure 5H), *Type IIA* (Figure 5I), and *Type IIX* (Figure 5K) all increased significantly compared with the ZEN group. Specifically, the mRNA expression of *Type I* (*p* < 0.05) and *Type IIA* (*p* < 0.05) in the ZEN + Melatonin group completely rescued the myofiber shift phenotype caused by ZEN (Figure 5H,I). Moreover, the ZEN + Melatonin group had significantly higher mRNA levels of *Type IIA* than the ZEN + GSH and ZEN + NMN groups (Figure 5I). Collectively, ZEN exposure affected muscle fiber composition, which was rescued by NMN, GSH, and melatonin in vivo, and melatonin had a better-rescued effect on ZEN damage.

### 3.5. ZEN Induces Slow-to-Fast Myofiber Shift Through Oxidative Stress and Mitochondrial Dysfunction

**Figure 6:**
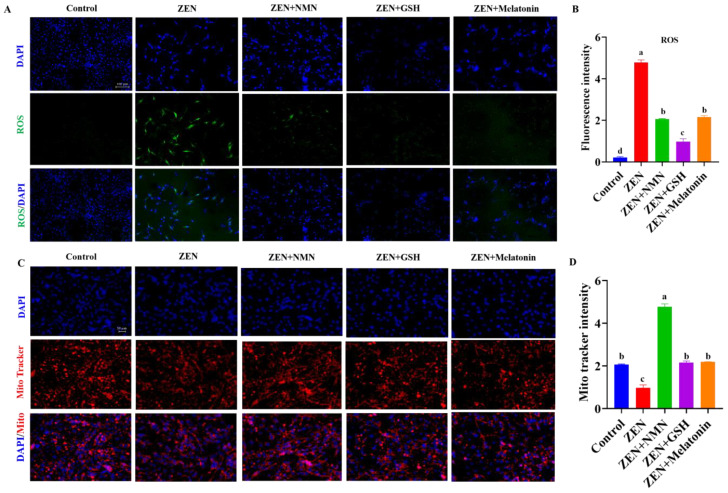
ZEN combined antioxidants affect muscle fiber transformation by mitochondria content and oxidative stress. (**A**,**B**) The ROS probe (**A**) (scale bar: 100 μm) and count analysis (**B**) of myoblasts in the aforementioned groups, (**C**,**D**) the images (**C**) (scale bar: 50 μm) and count analysis (**D**) of mito-tracker staining in myoblasts treated by ZEN or ZEN combined with antioxidant (NMN, GSH, and melatonin). Different letters (a–d) indicate significant differences (*p* < 0.05), and the same letters indicate no difference, *n* = 3.

ROS levels and mitochondrial content were detected to explore the mechanism of action of ZEN on myofibers further. The data showed that the fluorescence intensity of ROS levels in the ZEN group significantly increased compared with the control group, while the ROS levels significantly reduced after the treatment of NMN, GSH, and melatonin (Figure 6A,B). Thus, Mito-Tracker staining results showed that the mitochondrial content of the ZEN group was significantly reduced compared with the Control group, while the combined treatment of NMN, GSH, and melatonin in the ZEN group significantly restored the mitochondrial staining signal (Figure 6C,D). Further, the ZEN + GSH group significantly decreased the fluorescence intensity of ROS levels compared with the ZEN + NMN and ZEN + Melatonin groups (Figure 6B). The ZEN + NMN group had the strongest mitochondria staining signal compared with the ZEN + GSH and ZEN + Melatonin groups (Figure 6D). Therefore, ZEN exposure resulted in excessive ROS accumulation, mitochondrial dysfunction, and subsequent changes in myofiber composition. Antioxidants (NMN, GSH, and melatonin) alleviated these symptoms. Among the three antioxidants, NMN has a better ability to increase mitochondrial content and reduce oxidative stress.

### 3.6. ZEN Combined with Antioxidant Treatment Indirectly Affects Slow-to-Fast Myofiber Shift by Altering the Proportion of the Gut Microbiota

**Figure 7:**
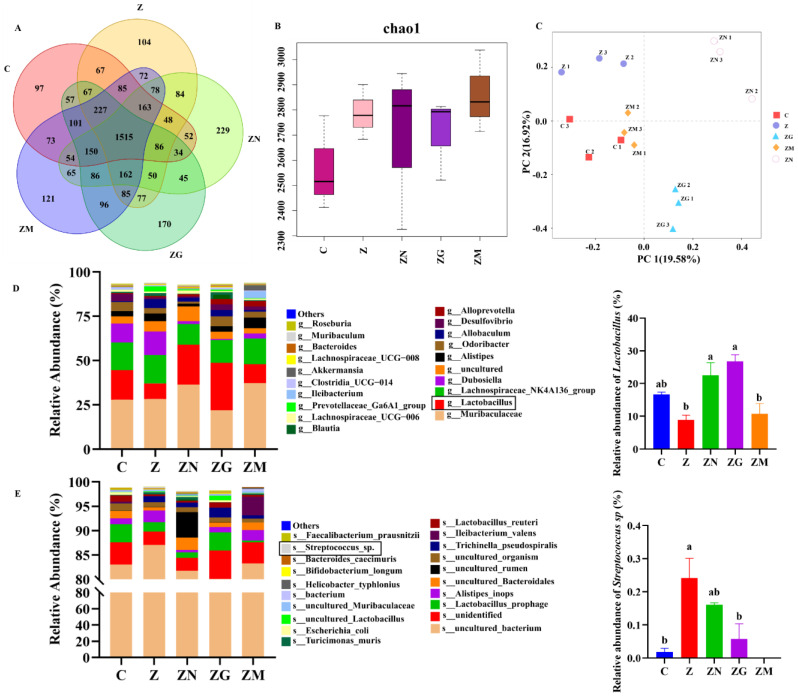
ZEN combined with antioxidants affects myofiber types through changes in the proportion of gut microbes. (**A**) Venn diagram of microbial OTUs in five groups (Z, ZEN group; ZN, ZEN + NMN group; ZG, ZEN + GSH group; ZM, ZEN + Melatonin group; C, Control group) of mice, (**B**) microbial richness (Chao1 index), (**C**) PCA plot of mice in five groups, (**D**,**E**) differences in the genus level (**D**) and species level (**E**) of gut microflora in the five groups of C57BL/6J mice. Different letters (a and b) indicate significant differences (*p* < 0.05), and the same letters indicate no difference, *n* = 3.

Previously, research found that the gut-muscle axis indirectly affects skeletal muscle mass by microbiota [29]. Thus, we performed 16S rDNA sequencing to explore the indirect effects of ZEN and antioxidants on skeletal muscle through gut microbiota composition. As shown in the Venn diagram, there were a total of 14,888 operational taxonomic units (OTUs) in these five groups (Figure 7A). Of which, 1515 OTUs overlapped in these five groups, and 97, 104, 229, 170, and 121 specific OTUs respectively existed in the control, ZEN, ZEN + NMN, ZEN + GSH, and ZEN + Melatonin groups (Figure 7A). The chao1 index was higher in the four treated groups than in the control group (Figure 7B). In the principal component analysis (PCA) of the first principal factor (19.58%) and the second principal factor (16.92%), these five groups of intestinal samples were divided into five obvious clusters, indicating that they had significant differences in intestinal microbial structure (Figure 7C). It was reported that feeding *Lactobacillus paracasei PS23* (LPPS23) and *Lactobacillus casei Shirota* (LcS) attenuates age-related sarcopenia in SAMP8 mice via the gut-muscle axis [30]. At the genus level, ZEN (8.88%) decreased the abundance of *Lactobacillus* compared with the control (16.62%) (Figure 7D and Table S1). Compared with the ZEN group, the addition of NMN (22.51%, *p* < 0.05), GSH (26.79%, *p* < 0.05), and melatonin (10.68%) increased the abundance of *Lactobacillus*, respectively (Figure 7D and Table S1). Furthermore, *Streptococcus* can produce lactic acid [31], and *Streptococcus pneumoniae* can affect glycolytic activity [32] and may be associated with fast-twitch fiber biogenesis [33]. Among the top 20 bacteria at the species level, the relative abundance of *Streptococcus* sp. in the ZEN group (0.24%) was significantly increased compared with the control group (0.02%). However, this kind of bacteria in the ZEN + NMN group (0.16%), ZEN + GSH group (0.06%, *p* < 0.05), and ZEN + Melatonin group were decreased compared with the ZEN group (Figure 7E and Table S2). These results suggested that the levels of the dominant genus of the gut microbiota in mice exposed to ZEN were altered, which might affect myofiber types.

## 4. Discussion

ZEN contamination has become a significant global threat to animal and human health. Some evidence indicates that ZEN-contaminated food fed to livestock and poultry (wild boars [10] and Hy-Line Brown laying hens [11], etc.) could be detected in their residues in muscle tissue. However, the exact damage of ZEN on skeletal muscle remains largely unknown. In this study, ZEN exposure in vitro decreased the mRNA levels of the proliferative marker genes (*CCND1*, *CCNB1*, and *CDK2*) and the myogenic marker genes (*MYHC*, *MYOD1*, *MYOG*, and *MYF5*) in myoblasts, but GSH, melatonin, and NMN reversed this effect. Similar to these results, studies have shown that the addition of 10 μmol/L and 30 μmol/L of ZEN to donkey granulosa cells (dGCs) and porcine GCs cultured for 72 h in vitro significantly reduced mRNA expression of the *CDK2* and *CCNB1* genes [34,35]. Furthermore, the literature showed that the expression of *MYOG* and *MYOD1* genes in zebrafish larvae exposed to 4 μmol/L ZEN was significantly decreased, and their development was defective [36]. Gao et al. found that Yorkshire sow feeding of ZEN-contaminated food reduced the expression of *MYF5* and *MYOD1* in offspring [13]. These results indicated that ZEN exposure inhibits the proliferation and myogenic differentiation of myoblasts.

Oxidative stress refers to the stimulation of organisms by harmful factors, resulting in the excessive production of ROS, which disrupts the balance of the antioxidant system, impairs cell function, and ultimately leads to apoptosis or necrosis [37,38]. We found that ZEN increased ROS levels, which causes oxidative stress and mitochondrial damage. Studies have shown that ZEN can induce ROS production and cause oxidative stress at different dosages [37,39]. Cao et al. found that 25 μmol/L ZEN increased ROS production, decreased antioxidant enzyme activity, and induced oxidative damage in Sertoli cells in piglets [40]. The toxicity of ZEN to cardiac cells (H9c2) is achieved by increasing ROS levels, loss of mitochondrial transmembrane potential (ΔΨm), and activation of caspase [41]. Furthermore, 10 μmol/L ZEN could induce oxidative stress in porcine embryos, increase reactive oxygen species (ROS) levels, and cause mitochondrial dysfunction to affect development, and melatonin could rescue the reproductive toxicity of ZEN to embryos [42]. Our results also showed that ZEN elevated ROS levels, decreased mitochondrial content, and caused mitochondrial swelling, but antioxidants attenuated the pathological symptoms. For the potential molecular mechanism of ZEN regulation on oxidative stress, Long et al. found that the sertoli cell TM4 of mice exposed to ZEN decreased the mRNA and protein levels of NF-E2-related factor-2 (Nrf2) and decreased the mRNA and protein levels of heme oxygenase 1 (HO-1), quinone oxidoreductase 1 (NQO1), γ-glutamylcysteine synthetase (γ-GCS), and glutathione peroxidase (GPX) in the downstream part of the Nrf2 signal pathway [20], which suggested that ZEN may inhibit the Nrf2/ARE signaling pathway, thus caused oxidative damage.

In this study, we found that ZEN produced oxidative stress, then reduced the average area and diameter of muscle fibers and exhibited the pathology of myolysis. Consistently, Yorkshire sows ingested ZEN-contaminated food, accompanied by pale sarcoplasm and optically empty vacuoles in the muscles, and even led to a decrease in myofiber diameter for the offspring [13]. Recently, Wang et al. found that zebrafish larvae treated with ZEN had a phenotype of reduced trunk myofibrils, reduced density, nuclei lysis, sarcoplasmic reticulum swelling, reduced distance of motion, and reduced mean velocity [36]. Except for these symptoms, ZEN exposure induced slow myofiber changes into fast myofiber in our work, but the addition of NMN, GSH, and melatonin restored this phenomenon. It was known that slow-twitch fibers’ proportion was positively correlated to meat quality, including color, tenderness, water-holding capacity, etc. [43]. The slow-to-fast twitch fiber shift might be explained by slow-twitch fibers being more susceptible to oxidative stress than fast-twitch fibers, which was verified in the prepubertal gilts’ slow-twitch fibers under ZEN exposure, increasing malondialdehyde (MDA) concentration and decreasing GPX-1 protein abundance and activity, but not in the fast-twitch myofibers [44]. In contrast, mice increased the mRNA and protein levels of Nrf2 and increased the protein levels of NQO1 and HO-1, thereby enhancing antioxidant capacity and inducing the fast-to-slow twitch fibers shift [45]. In this current work, we preliminarily explore the damaging effect of ZEN on muscle fibers caused by oxidative stress (increasing ROS levels and decreasing mitochondrial count), but the pathways (signaling pathways or cellular processes) through which ZEN affects have not yet been explored. It will be the focus of our future work.

Generally, the metabolism of ZEN and its derivatives in animals is through the liver and gastrointestinal transformation pathways and is finally excreted through feces and urine [46]. However, several works showed that ZEN and its metabolites were detected in the muscle tissues of pigs (hybrids of *Deutsches Edelschwein* and *Pietrain*) [46] and wild boar (*Sus scrofa*) [47] fed with ZEN-contaminated food. Specifically, the mean concentrations of ZEN in the liver, muscle, and kidney samples of wild boar (*Sus scrofa*) were 1.71 ng/g, 1.49 ng/g, and 0.65 ng/g, respectively, and the mean α-zearalenol values were 0.65 ng/g, 0.66 ng/g, and 0.77 ng/g, respectively. In pigs (hybrids of *Deutsches Edelschwein* and *Pietrain*) muscle tissue, zeranol with concentrations of up to 13.3 μg/kg along with α-zearalenol (up to 14.5 μg/kg) and traces of ZEN and taleranol were detected. This suggested that ZEN and its metabolites were able to accumulate in muscle tissue, and muscle was also a toxicity target tissue of ZEN. Currently, our work has found that ZEN direct treatment inhibited myogenic differentiation and changed the myofiber compositions in vitro, which indicated that ZEN at least partly causes these effects. However, we still cannot exclude the effect of its metabolites contributing to these phenotypes, and their contributions will be explored in a further study.

Beyond the direct effect of mycotoxin (such as ZEN) on skeletal muscle, mycotoxin also disrupts the intestinal flora [48], which influences bacterial community diversity, involving muscle function and development through the gut-muscle axis and leading to impairing bodily functions and ultimately reducing the quality of life [49]. Studies have shown that Lactobacillus strains (*Lactobacillus fermentum DR9*, *Lactobacillus paracasei OFS 0291*, and *L. helveticus OFS 1515*) protected the skeletal muscle from aging through antioxidant effects, of which *Lactobacillus fermentum DR9* had the strongest regulatory role [50]. We found that the results of 16S rDNA sequencing of cecal contents in this study showed that ZEN treatment decreased the relative abundance of *Lactobacillus* at the genus level, and ZEN + NMN and ZEN + GSH treatment groups rescued its abundance. In addition, at the species level, the relative abundance of *Streptococcus* sp. in the ZEN group was higher than that of the Control group, which was reported to produce lactic acid [51]. The limitation was that the exact roles of gut microbiota alteration induced by ZEN and antioxidant regulation on myofiber composition need to be further studied. Studies have shown that the small intestine of Merino Wethers can absorb lactic acid, which then enters the blood [52]. Furthermore, plasma lactate can affect muscle metabolic performance and muscle mass [53]. Compared with slow-twitch fibers, fast-twitch fibers facilitate the production of lactic acid through the glycolytic pathway [32,33]. However, whether lactic acid produced by *Streptococcus* sp. affects the composition of muscle fibers would be the focus of our future work.

In this study, ZEN exposure increases the level of ROS and damages mitochondria to cause oxidative stress, subsequently affecting the composition of myofibers. Interestingly, antioxidant gavage (NMN, GSH, and melatonin) all exhibited a protective effect on ZEN-damaged skeletal muscle to different extents. It was reported that NMN oral administration prevents doxorubicin-induced cardiotoxicity and skeletal muscle loss in mice by defending against oxidative stress, indicating by enhancing NAD^+^ turnover and decreasing the accumulation of 4-hydroxy-2-nonenal (4-HNE), a biologically active aldehyde of lipid peroxidation [54]. Consistently, NMN rescued the ZEN-induced side effect of skeletal muscle also through the alleviation of oxidative stress, manifested by the reduction of ROS level and increasing mitochondrial content. However, the potential molecular mechanism of NMN against oxidative stress in skeletal muscle induced by ZEN needs further exploration.

GSH is an endogenous antioxidant, and the salvage mechanism of exogenous GSH addition to damaged skeletal muscle is still unclear. The content of GSH in mice skeletal muscle stem cells (MuSCs) changed with age, and a high content of endogenous GSH increased the expression of *NRF2* by increasing the total transcript levels of microsomal glutathione S-transferase 1 (Mgst1), as an endoplasmic reticulum glutathione transferase and peroxidase, increased mitochondrial turnover to decrease mitochondrial ROS and promote myogenesis [55]. Similarly, the exogenous addition of GSH rescued the proliferation of myoblasts in ZEN-injured skeletal muscle in our study, but whether GSH rescued damage to skeletal muscle induced by ZEN through the NRF2 signaling pathway needs further study. For melatonin, 50 mg/kg BW partly alleviated the muscle damage induced by carbon tetrachloride (CCl_4_) [56]. In our work, myolysis pathology was not observed in the ZEN+ Melatonin group, which was also associated with the promotion of fast-to-slow myofiber shift. However, the molecular mechanism of melatonin rescue ZEN inducing myolysis and the shift in slow-to-fast twitch fibers still need to be further explored.

## 5. Conclusions

**Figure 8:**
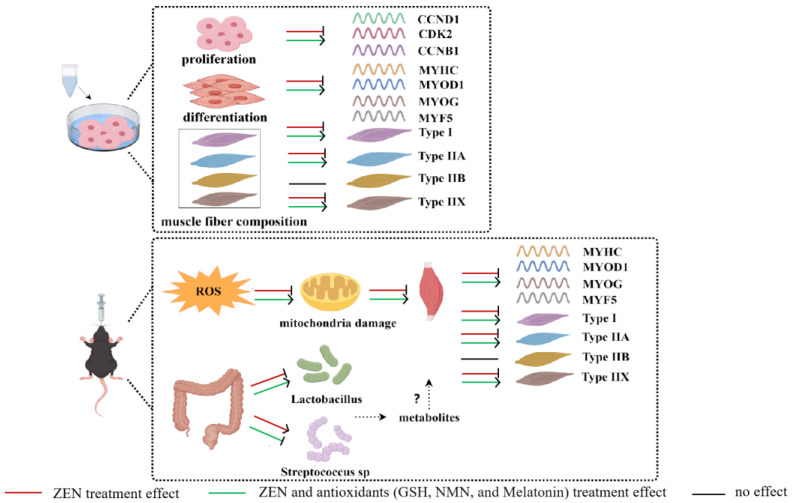
A schematic diagram of ZEN damage to skeletal muscle in vitro and in vivo and salvage of 3 antioxidants (NMN, GSH, and melatonin) was drawn by “Figdraw 2.0” software.

Summarily, we found that ZEN inhibits myoblast proliferation and differentiation in vitro, and antioxidants (NMN, GSH, and melatonin) rescue these effects. In addition, ZEN exposure induced slow-to-fast myofiber shift in vivo and in vitro, which was restored by antioxidant treatments. Pathologically, ZEN led to ROS production and oxidative stress, and subsequently, pathological symptoms occurred, including mitochondrial dysfunction, myolysis, etc., which were combated by antioxidant treatment. Moreover, ZEN and antioxidants might indirectly affect muscle fibers via a reduced relative abundance of *Lactobacillus* and an increased relative abundance of *Streptococcus* sp., respectively (Figure 8). Collectively, this study explores the pathology of ZEN on muscle and the salvage roles of NMN, GSH, and melatonin for ZEN toxicity, which would provide new insights and promising attenuation solutions for ZEN-damaging skeletal muscle. However, the underlying molecular mechanism of ZEN and the 3 antioxidants (NMN, GSH, and melatonin) in the skeletal muscle still needs to be explored.

## Figures and Tables

**Table 1:** RT-qPCR primer sequences.

Gene	Forward Primer (5′–3′)	Reverse Primer (5′–3′)	Length (bp)	Gene Accession Number
*MYHC*	GCGAATCGAGGCTCAGAACAA	GTAGTTCCGCCTTCGGTCTTG	138	XM_017314318.3
*MYOG*	GAGACATCCCCCTATTTCTACCA	GCTCAGTCCGCTCATAGCC	106	NM_031189.2
*MYF5*	AAGGCTCCTGTATCCCCTCAC	AAGGCTCCTGTATCCCCTCAC	213	NM_175686.3
*RPL7*	TGGTTTAGGAGAGTAAGGTTGCT	TGGTTTAGGAGAGTAAGGTTGCT	351	XM_006529040.3
*MYOD1*	CCACTCCGGGACATAGACTTG	AAAAGCGCAGGTCTGGTGAG	109	NM_010866.2
*CCND1*	TAGGCCCTCAGCCTCACTC	CCACCCCTGGGATTGGTTTA	338	XM_006529043.2
*CCNB1*	CTTGCAGTGAGTGACGTAGAC	CCAGTTGTCGGAGATAAGCATAG	94	NM_172301.3
*CDK2*	CAAAGCCAAGCACGTAGAGAC	TGCACCACATATTGACTGTCC	141	NM_053180.2
*MYH7*	GAATGGCAAGACGGTGACTGTG	GAAGCGTAGCGCTCCTTGAG	233	gi|1698894
*MYH2*	ATCAACCAGCAGCTGGACACCA	TCCAGCACGAACATGTGGTGGT	249	gi|5360745
*MYH4*	ACAGACTAAAGTGAAAGCCTACAA	CACATTTTGTGATTTCTCCTGTCAC	257	gi|5360749
*MYH1*	CCAATGAAACCAAGACTCCTGG	TGCTATCGATGAACTGTCCCTC	234	gi|5360747

## Data Availability

The data that support the findings of this study are available from the corresponding author upon reasonable request. The raw data supporting the findings of this study are available from the corresponding authors upon reasonable request. The 16S rDNA sequencing raw data during this study were submitted to the National Center of Biotechnology Information (No. PRJNA1173076).

## Supplementary Materials

The following supporting information can be downloaded at: https://www.mdpi.com/article/10.3390/antiox14050528/s1.

## Abbreviations

CCND1	Cyclin d1
CCNB1	Cyclin b1
CDK2	Cyclin-dependent kinase 2
MYHC	Myosin heavy chain
MYOD1	Myogenic differentiation 1
MYOG	Myogenin
MYF5	Myogenic factor 5
TA	Tibialis anterior
GAS	Gastrocnemius muscle
EDL	Extensor digitorum longus
SOL	Soleus
DAPI	4′,6-diamidino-2-phenylindole
ROS	Reactive oxygen species
DCFH-DA	2′,7′-dichlorodihydrofluorescein diacetate
TEM	Transmission electron microscopy
